# A Bioelectric Active Hydrogel Sensor for Trace Detection of Heavy Metal Ions in Livestock and Poultry Farm Wastewater

**DOI:** 10.3390/bios15060341

**Published:** 2025-05-29

**Authors:** Heng-Chi Liu, Jia-Xin Du, Jie Wang, Junying Liu, Luyu Yang, Yang-Chun Yong

**Affiliations:** Institute for Energy Research, School of Environment and Safety Engineering, Jiangsu University, 301 Xuefu Road, Zhenjiang 212013, China; hengchiliu2003@163.com (H.-C.L.); 14729257987@163.com (J.-X.D.); w1444178983@163.com (J.W.); lsliujy@ujs.edu.cn (J.L.)

**Keywords:** livestock and poultry wastewater, microbial surface display, graphene hydrogel, heavy metal, sensors

## Abstract

Heavy metal contamination in livestock and poultry farm wastewater poses significant risks to both the environment and human health, so it is critical to accurately and rapidly quantify heavy metal ion concentrations in water. This research develops a bioelectric active hydrogel sensor for detecting heavy metal ions in livestock wastewater. The sensor integrates microbial surface display technology with graphene hydrogel, displaying glucose oxidase (GOx) on the surface of yeast cells, and covalently incorporating it into the graphene hydrogel through the bio-reduction activity of metal-reducing bacteria, enhancing its electrochemical performance. The sensor demonstrates excellent sensitivity and stability in detecting Cu^2+^, with a detection limit for Cu^2+^ of 17.0 µM. This sensor is also applicable for detecting Zn^2+^ in wastewater. When various heavy metal ions coexist in the solution, they exert a more pronounced inhibitory effect on enzyme activity. Consequently, the sensor can be employed to assess the overall heavy metal content in water samples. In the detection of Cu^2+^ in real livestock and poultry wastewater, the recovery rate of the graphene hydrogel electrode ranged from 88% to 106.5%, indicating that the sensor holds significant potential for application in actual sample analysis.

## 1. Introduction

Heavy metal ions (e.g., copper ions) in livestock and poultry farming wastewater may pose serious threats to aquatic ecosystems and human health at high concentrations [1,2,3]. Due to the persistence and stability of heavy metals, these pollutants are widely distributed across various environmental media—including water, air, and soil, and are resistant to natural degradation [4,5]. Excessive accumulation of heavy metals can severely affect the health of organisms. For example, an overabundance of mercury can impair brain tissue and the nervous system [6], while excessive cadmium can damage the liver and kidneys [7,8], Chromium can induce protein denaturation and disrupt enzymatic activity [9]. According to the Chinese National Standard (GB 8978-1996) [10], the discharge of wastewater from livestock and poultry farms must comply with the total copper (Cu) ≤ 2.0 mg L^−1^ and total zinc (Zn) ≤ 5.0 mg L^−1^ discharge limits. Even heavy metals with relatively low toxicity, such as copper, may pose health risks when ingested in excess [11,12]. In view of this, the detection and control of heavy metal pollution are essential steps in protecting the ecological environment, which in turn contributes to the broader goal of promoting a harmonious relationship between humans and nature [13,14]. Although traditional heavy metal detection methods, such as atomic absorption spectroscopy (AAS), colorimetric method, and inductively coupled plasma mass spectrometry (ICP-MS), are highly sensitive, they generally require complex sample pretreatment, involve expensive instrumentation, and are not conducive to rapid on-site analysis [15,16]. Therefore, developing a simple, efficient, portable, and sensitive sensor for the detection of heavy metal ions in livestock and poultry wastewater is extremely urgent.

In recent years, with continuous advancements in enzyme engineering and electrode materials, enzyme-based novel biosensors have gradually emerged as a research hotspot [17,18]. In particular, in the field of heavy metal ion detection, enzyme-based biosensors have demonstrated considerable application potential owing to their exceptional sensitivity and specificity [19,20]. These sensors typically harness the interactions between enzymes and heavy metal ions to induce electrochemical changes, which can be accurately detected using electrochemical instrumentation [21,22]. For instance, an amperometric enzyme-based electrochemical sensor was developed by immobilizing GOx on poly (o-phenylenediamine), and various heavy metal ions, including Hg^2+^, Ag^+^, Cu^2+^, Cd^2+^, Pb^2+^, and Cr^3+^ were successfully detected [23]. GOx was immobilized onto an electrode modified with ZnO nanorods, and the inhibitory effect of Hg^2+^ on the enzyme was exploited for heavy metal ion detection, yielding a detection limit for Hg^2+^ of 0.5 nM [24]. A biosensor based on screen printing that utilizes urease for the detection of silver and copper was developed [25]. Biochemical methods exhibit high sensitivity and ease of miniaturization, making them highly suitable for on-site monitoring operations. However, purifying the enzyme and immobilizing the electroactive enzyme onto the electrode is a cumbersome process, and with prolonged use, the enzyme activity on the electrode diminishes, resulting in poor reproducibility and stability of the sensor [26,27]. Therefore, enzyme-based electrochemical sensors still face issues and challenges in both large-scale production and complex applications.

Microbial surface display technology, as a cutting-edge bioengineering tool, holds promise for providing new opportunities for the further development of enzyme-based electrochemical sensors [28,29,30,31]. This technology utilizes the surface of biological cells as a platform for the display of exogenous proteins, achieved by fusing exogenous proteins with carrier protein genes. The carrier protein facilitates translocation across the cell membrane and anchors the exogenous proteins onto the bacterial cell surface. For instance, the OmpC protein was successfully employed as an anchoring motif to display lipase on the surface of *E. coli*, with the enzyme’s activity further enhanced by gene co-expression [32]. OmpA was employed to display peptide segments on the surface of fluorescent *Escherichia coli*, thereby achieving high-throughput screening for red blood cell binding [33]. Microbial surface display technology has paved multiple avenues for diverse applications of surface display, thereby enhancing the efficiency of protein expression and purification processes. By directly displaying active proteins or enzymes on the bacterial surface, this approach has demonstrated extensive application potential in the detection of heavy metal ions [34,35,36].

**Scheme 1:**
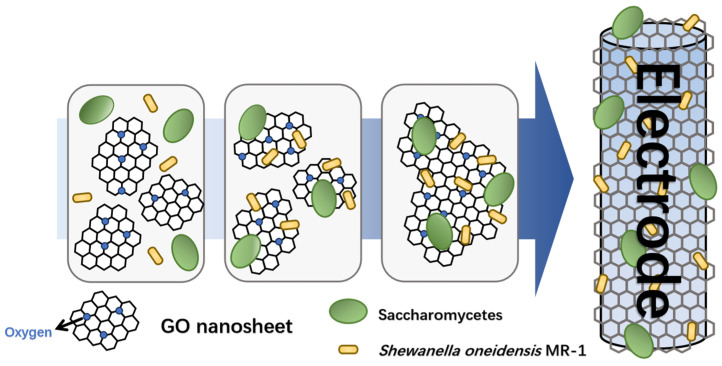
Construction diagram of bioelectrically active hydrogel electrode.

Herein, a graphene hydrogel with electroactive enzyme was constructed by combining microbial surface display technology with graphene-based hydrogel self-assembly. The formation of hydrogel is attributed to the capture of free bacteria by graphene oxide (GO) nanosheets, and the formation of an electroactive enzyme hydrogel electrode (Scheme 1) on the titanium wire in the capillary through the reduction of *S. oneidensis* MR-1. Compared to existing graphene-based biosensors, our integrated approach of microbial surface display and graphene hydrogel dispenses with enzyme purification and enables efficient electron transfer without complex modifications to the electrode. This sensor exhibits excellent electrochemical performance with significantly improved minimum detection limits and cycling stability, providing the basis for the electrochemical detection of heavy metal ions. A bioelectric activity sensor was developed based on the inhibitory effect of heavy metals on enzymes, achieving the detection of trace amounts of heavy metal ions (Cu^2+^, Zn^2+^) in samples. This method has been successfully applied to detect heavy metal ions in livestock wastewater, offering an innovative tool for monitoring heavy metal pollution and presenting potential solutions for environmental remediation.

## 2. Materials and Methods

### 2.1. Materials

All reagents were purchased from Sinopharm Chemical Reagent Co., Ltd. (Shanghai, China) unless otherwise indicated. LIVE/DEAD^®^ BacLight™ Bacterial Viability Kit was obtained from Thermo Fisher Scientific (Waltham, MA, USA). All the agents are obtained from commercial suppliers and used as received unless otherwise specified.

### 2.2. Strain Culture Condition

*S. oneidensis* MR-1 strains were preserved at −70 °C and cultured in LB medium (10 g/L NaCl, 10 g/L tryptone, 5 g/L yeast extract, pH 7.0) in a constant temperature incubator set at 30 °C, with shaking at 180 rpm. The EBY 100 (pYD1-GOx) strain was cultured by picking a single colony into YNB-CAA medium (6.7 g/L YNB without amino acids, 5 g/L casein hydrolysate, 20 g/L glucose) and incubating at 30 °C with shaking at 180 rpm overnight. When the OD_600_ reached 2–5, cells were collected by centrifugation at 4000× *g* for 5 min, resuspended in YNB-CAA induction medium (6.7 g/L YNB without amino acids, 5 g/L casein hydrolysate, 20 g/L galactose) to an OD_600_ of 0.5–1, and induced at 20 °C for 20 h with shaking at 180 rpm.

### 2.3. Experimental Material Pretreatment

Pretreating the working electrode titanium wire, counter electrode platinum wire, and reference electrode silver wire by removing surface impurities with acetone and hydrochloric acid. Preparing the capillary glass tube by heating it with an alcohol lamp to form a tip and then ultrasonically treating it.

Prior to use, the carbon cloth and carbon felt were sequentially treated by: (1) immersion in acetone for 12 h to remove surface organics; (2) soaking in 2 M HCl for 4 h to eliminate inorganic impurities; and (3) thorough rinsing with deionized water via ultrasonication. This cleaning cycle (HCl treatment followed by DI water rinsing) was repeated three times, with final neutralization confirmed by pH testing. The substrates were then dried at 60 °C for subsequent experiments.

### 2.4. Preparation of Capillary Hydrogel Electrodes

Take appropriate volumes of Shewanella and yeast broth with a final OD_600_ of 3 into centrifuge tubes. Centrifuge at 5000 rpm for 5 min, discard the supernatant, washed three times with sterile water, and resuspend the bacterial cells in 500 mL of culture medium (16.95 g/L Na_2_HPO_4_·12H_2_O, 2.85 g/L KH_2_PO_4_, 0.95 g/L NaCl, 0.975 g/L NH_4_Cl, 1 g/L tryptone, 0.5 g/L yeast extract, 0.1 mol/L CaCl_2_, 1 mol/L MgSO_4_·7H_2_O). Preparing a graphene hydrogel solution by adding 250 mL of GO and 250 mL of pure water, resulting in a final GO concentration of 1 mg/mL in the system. The bacterial suspension was adjusted to a final OD_600_ = 3 in the 1 mL system (500 µL concentrated culture + 250 µL GO + 250 µL water). The required volume of pre-culture was calculated based on its measured OD_600_ (X) using: V₁ = (3 × 1000 µL)/X. After centrifugation (5000 rpm, 5 min) of V₁ volume, the pellet was resuspended in 500 µL fresh medium to achieve the target OD_600_. Inject 60 mL of the hydrogel solution into a capillary. Placing a titanium wire of suitable length into the capillary as the working electrode, secure the titanium wire with parafilm, and incubate at 30 °C for 20–24 h to form the graphene hydrogel.

### 2.5. Characterization of Hydrogel Cell Activity

Using the LIVE/DEAD^®^ BacLight™ Bacterial Viability Kits L7012 to characterize hydrogel cell activity. This kit contains two reagents, SYTO 9 and PI. Here are the operation steps: First, prepare the dyes according to the kit instructions and store them at −20 °C in the dark. Next, centrifuge and wash the graphene hydrogel three times with 0.85% physiological saline to remove the culture medium. Then, put a small piece of the hydrogel sample into an EP tube, pipette 50 μL of the dye into it, and let it stand in the dark for about 15 min. Finally, pipette an appropriate amount of the solution onto a glass slide and conduct confocal laser scanning microscopy (CLSM) observation.

### 2.6. Construction of Bioelectrochemical Test Platform

After the cultivation of the capillary hydrogel is completed, suck out the solution inside the capillary hydrogel and add PBS buffer (containing 0.5 M glucose). Insert a platinum wire (counter electrode) and a silver wire (reference electrode) into the capillary respectively. Seal and fix the capillary tube opening. Connect the three-electrode system to a CHI660E electrochemical workstation to complete the construction of the bioelectrochemical testing platform.

### 2.7. Electrochemical Detection of Glucose and Heavy Metal Ions

Differential pulse voltammetry (DPV) is used to detect glucose and heavy metal ions. The detection system was connected to the electrochemical workstation as described in Section 2.6, and the scanning range is from −0.7 V to −0.2 V, with an amplitude of 0.05 V, a pulse period of 0.5 s, and a pulse width of 0.05 s. The tip of the capillary is used to aspirate a sample of PBS buffer solution containing 0.5 M glucose. A differential pulse voltammetric scan is then performed to obtain a current curve. The height values of the resulting current peaks are calculated and recorded.

### 2.8. Reusable Performance Test

The one cycle of the reusable stability test consists of the following standardized procedure: after the detection of heavy metal ions by the capillary hydrogel sensor, the electrolyte is replaced with a 1 M EDTA solution (chelating agent) and then left to incubate for 15 min to completely remove the heavy metal ions. Finally, the current response and inhibition effect of the solution were remeasured after replacing the electrolyte solution with a new one and adding fresh Cu^2+^ (same concentration).

## 3. Results

### 3.1. Design of a GOx Surface Display System on Saccharomyces Cerevisiae (EBY100)

**Figure 1:**
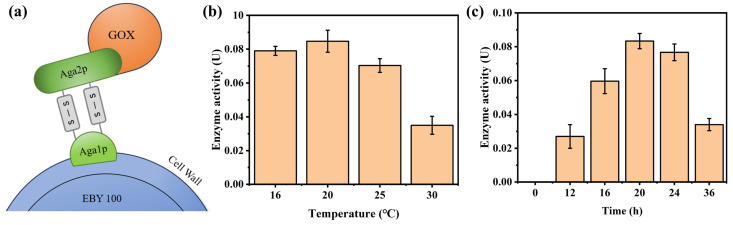
(**a**) Schematic representation of the yeast surface display system GOx; (**b**) Optimization of different induction temperature conditions (induction time of 24 h); (**c**) Optimization of different induction time conditions (induction temperature of 20 °C).

As shown in Figure 1a, the surface display of GOx was achieved using the a-agglutinin system of Saccharomyces cerevisiae (EBY100). After fusing GOx with the Aga2p subunit, it is linked to Aga1p via two pairs of disulfide bonds, thereby enabling the expression of GOx on the yeast surface. The GOx gene sequence was synthesized into the pYD1 plasmid via gene synthesis by Shanghai Shengong Company, resulting in the pYD1-GOx recombinant plasmid and the subsequent acquisition of the engineered *E. coli* Top10 (pYD1-GOx) strain. The plasmid map of the constructed plasmid is shown in Figure S1. The validation results after digestion with the restriction endonucleases Q.cut PstI and Q.cut NcoI are shown in Figure S2. The two bands, approximately 1500 bp and 5000 bp respectively, match the sizes of the corresponding fragments of the recombinant plasmid (1591 bp + 4960 bp), thereby confirming the successful construction of the recombinant plasmid (Figure S3). Using electroporation, the recombinant plasmid pYD1-GOx was further introduced into yeast-competent cells to construct the engineered strain EBY100 (pYD1-GOx). Plasmids were subsequently extracted from the engineered strain EBY100 (pYD1-GOx) using a yeast plasmid extraction kit, and PCR followed by agarose gel electrophoresis confirmed the successful introduction of the pYD1-GOx plasmid into the yeast cells. Subsequently, GOx expression was induced in the engineered strain EBY100 (pYD1-GOx). Figure 1b shows the variation in GOx surface display activity under different induction temperatures. Results indicate that when the induction temperature was 20 °C, the enzyme exhibited the highest activity at approximately 0.084 U, exceeding the activities observed at 16 °C (0.079 U) and 25 °C (0.07 U). At 16 °C, yeast growth and protein synthesis are slower, reducing the overall GOx expression level. At 20 °C, the balance between expression rate and proper folding is achieved, maximizing functional enzyme yield. At an induction temperature of 30 °C, the enzyme activity was the lowest, at only about 0.035 U. This may be attributed to the fact that at elevated temperatures, the bacterial growth rate increases, leading to a substantial accumulation of metabolites during fermentation, which in turn inhibits both the expression and proper folding of the target protein. Figure 1c presents the results of GOx surface display activity changes at various induction times. Results indicated that the activity of GOx initially increased with induction time and then decreased, with the highest activity (approximately 0.084 U) observed after 20 h of induction. Accordingly, an induction temperature of 20 °C and an induction time of 20 h were selected as the optimal conditions for GOx surface display.

### 3.2. Fabrication of the Bioelectric Activity Sensor

**Figure 2:**
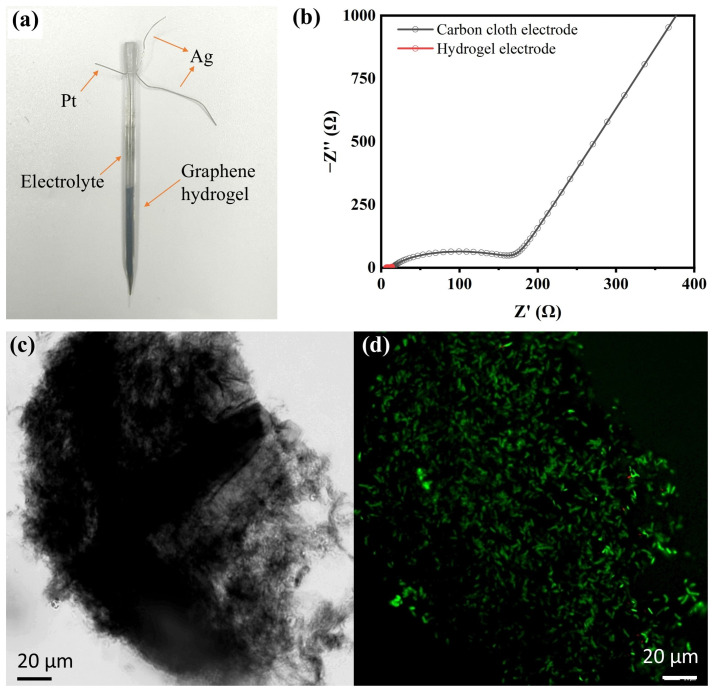
(**a**) Schematic diagram of the capillary hydrogel electrochemical detection platform; (**b**) Nyquist spectra of capillary hydrogel electrode and carbon cloth electrode; (**c**) Micrograph of hydrogel electrode; (**d**) CLSM image after hydrogel staining in the capillary hydrogel electrochemical detection platform.

Figure S4 illustrates the transformation process of the graphene hydrogel within the capillary. The solution’s color in the capillary changed from light brown at 0 h to black at 24 h due to the reduction of GO (C/O = 0.96) to rGO (C/O = 2.0)in the system. After 24 h, a clear phase separation between the upper portion of the hydrogel and the solution was observed, and the hydrogel at the capillary tip noticeably shrank, leaving a gap filled with solution, indicating the successful fabrication of the capillary graphene hydrogel electrode. The water content of the graphene hydrogel is about 91.54%. Subsequently, a three-electrode detection system was constructed within the capillary. As shown in Figure 2a, the graphene hydrogel served as the working electrode, a platinum wire as the counter electrode, and a silver wire as the reference electrode. The platinum and silver wires were fixed in the upper half of the capillary, while the graphene hydrogel occupied the lower half, and the positions of the three electrodes were adjusted to avoid mutual contact. Electrochemical impedance spectroscopy (EIS) results indicate that the Nyquist plots of the different electrodes display a near-semicircular shape in the high-frequency region and a straight line in the low-frequency region. The hydrogel electrode exhibited an impedance of 3.1 Ω (Figure 2b), which is over 50 times lower than that of the carbon cloth electrode (160 Ω). This indicates a significantly enhanced electron transfer capability among the electrode, the enzyme, and the biological components in the hydrogel system. Results indicate that graphene hydrogel exhibits excellent electrochemical activity, which facilitates electron exchange between the enzyme and the electrode, thereby enhancing the electrochemical performance of the enzyme-based bioelectric activity sensor.

Separate samples of the solution before hydrogel synthesis and the supernatant after synthesis were collected. After ultrasonic disruption, the protein content in these solutions was determined using a BCA protein assay kit. The protein content in the supernatant accounted for only 4.5–5.71% of the total protein, while the bacteria within the hydrogel comprised over 94% of the total, indicating the successful synthesis of the hydrogel and the efficient loading of both the bacteria and GOx within the capillary electrode. In Figure 2c, a large number of bacteria can be observed within the sheet-like graphene, demonstrating the high biological loading capacity of the hydrogel electrode. Figure 2d presents the fluorescence analysis results (green fluorescence indicates live cells and red fluorescence indicates dead cells). The results reveal that green fluorescence accounts for over 98% of the total, with the number of live cells far exceeding that of dead cells, demonstrating the excellent biocompatibility of the capillary hydrogel. In addition, the activity of GOx displayed on the graphene hydrogel was evaluated after the hydrogel was resuspended through vortexing. The results indicated that, compared to before hydrogel formation, the enzyme activity decreased by approximately 16%, with GOx maintaining relatively high activity. This underscores the advantages of microbial surface display. In summary, the capillary hydrogel electrode not only loaded a large number of bacteria but also maintained a high level of biological activity in the cells, with minimal loss of GOx enzyme activity.

### 3.3. Response Performance of the Bioelectric Activity Sensor to Heavy Metal Ions

**Figure 3:**
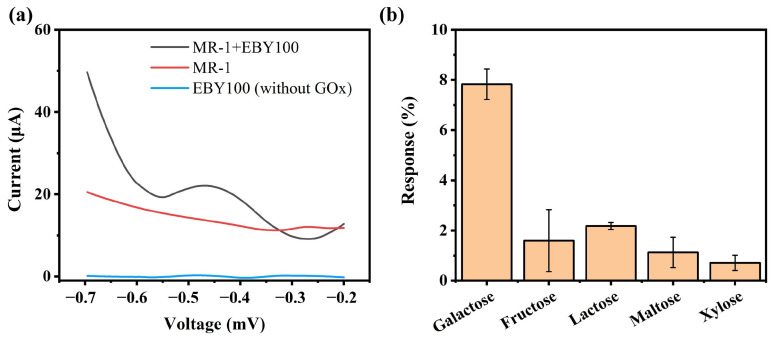
(**a**) Detection results of glucose by different sensors; (**b**) Specificity of the bioelectric activity sensor’s response to carbohydrates.

First, differential pulse voltammetry (DPV) was used to electrochemically detect the glucose response behavior of the bioelectroactive hydrogel sensor to verify the feasibility of the sensor. Figure 3a shows the DPV detection results of glucose solutions using different capillary hydrogel sensors. It can be observed that the hydrogel electrodes formed by the engineered strain EBY100 and MR-1 exhibited a distinct oxidation peak near −0.4 V, which is related to the oxidation of glucose by GOx on the electrode. In contrast, no noticeable oxidation peak was observed for the sensor constructed with MR-1 and the uninduced engineered strain. The specificity of the sensor was also analyzed. Common monosaccharides and disaccharides, including galactose, fructose, lactose, maltose, and xylose, were used as interfering substances. The same electrochemical detection method was applied to measure the oxidation current peak at −0.4 V, and the interference effects on the detection were analyzed by evaluating the size of the oxidation current peak. The results, shown in Figure 3b, indicate that the oxidation current peaks for galactose, fructose, lactose, maltose, and xylose near −0.4 V were 7.8% to 0.7% of the current peak observed for glucose detection. This demonstrates the enzyme’s substrate catalytic specificity and also confirms the feasibility of using bioelectroactive hydrogel-based sensors for electrochemical detection.

**Figure 4:**
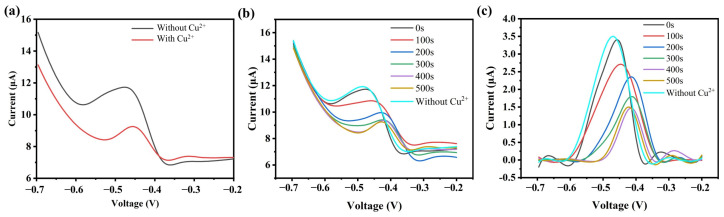
(**a**) Detection results of the bioelectric activity sensor before and after adding Cu^2+^; (**b**) Detection results of the sensor at different time points after adding Cu^2+^; (**c**) Analysis results after subtracting the background current.

Cu^2+^ was added to the capillary to evaluate the sensitivity of the response of the bioelectric activity sensor to heavy metal ions (Figure S5). As shown in Figure 4a, after adding a high concentration of Cu^2+^ to the capillary, the DPV current value significantly decreased (the maximum catalytic current near −0.45 V was 11.71 μA and 9.24 μA, respectively), and the area of the current peak also correspondingly decreased. The results indicate that Cu^2+^ exerts a significant inhibitory effect on GOx, suggesting that the capillary hydrogel electrochemical detection platform can not only detect glucose concentration but also be applied for the detection of heavy metal ions.

Further investigation was conducted to study the effect of the duration of Cu^2+^ addition on the detection performance during the static incubation period. Figure 4b shows the DPV detection results at different static incubation times after adding 5 mM of Cu^2+^. Baseline currents in pure support electrolytes (glucose, PBS) were recorded under the same experimental conditions, and DPV data without background current (Figure 4c) were obtained by subtracting the background current. The results indicate that when DPV scanning is performed immediately after adding Cu^2+^ to the capillary, the detection results are very similar to those of the samples without Cu^2+^. This may suggest that at this point, Cu^2+^ has not yet fully diffused into the capillary or has not sufficiently interacted with the active sites of the enzyme. As the incubation time increased, the inhibitory effect of Cu^2+^ on the current became more pronounced. After adding Cu^2+^ and incubating for 400 s, the current suppression rate of the sensor reached 57.8%, which is similar to the suppression rate of 58.1% observed after 500 s of incubation. After 500 s of incubation, the current suppression rate stabilized, likely because Cu^2+^ ions had fully diffused into the capillary and bound to the active sites of the enzyme, resulting in the maximum inhibitory effect. Therefore, this study used 400 s as the static incubation time after the heavy metal solution was introduced.

**Figure 5:**
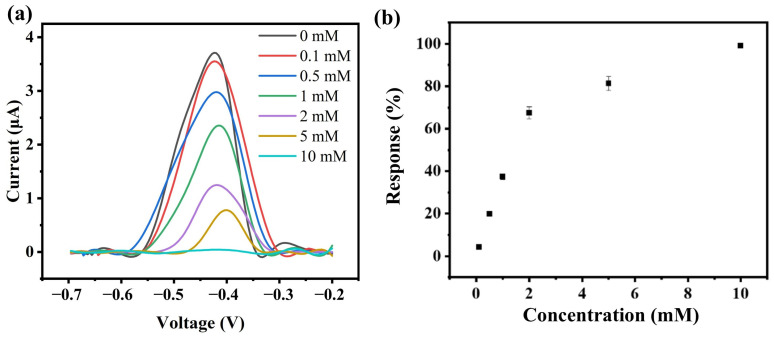
(**a**) Detection results of the sensor for different concentrations of Cu^2+^; (**b**) The linear range of the sensor calculated from (**a**).

Subsequently, the inhibitory effect of different concentrations of Cu^2+^ on the current was investigated. As shown in Figure 5a, the maximum current value decreased with increasing concentrations of Cu^2+^. At a Cu^2+^ concentration of 10 mM, the current was almost completely suppressed, while at a Cu^2+^ concentration of 5 mM, the current suppression rate was approximately 81.3%. A good linear relationship between Cu^2+^ concentration and the current suppression rate was observed when the Cu^2+^ concentration was below 2 mM. As shown in Figure 5b, a standard curve was prepared according to the concentration and inhibition of 0.1~2 mM Cu^2+^, and the lowest detection limit (LOD) (S/N = 3) of the sensor was calculated to be 17 μM. Compared to the typical detection methods reported (Table S1), this bioelectrorically active hydrogel-based sensor has shown comparable or better performance in the identification of heavy metal ions.

### 3.4. Stability of the Bioelectric Activity Sensor

**Figure 6:**
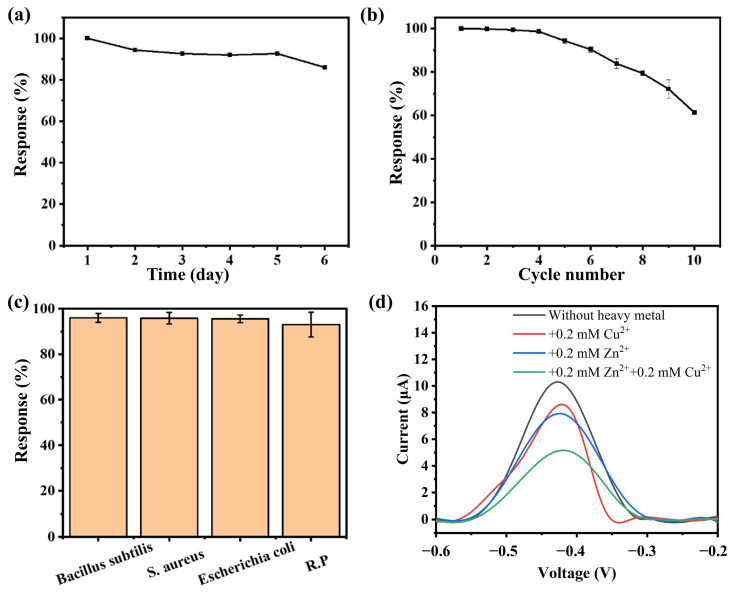
Long-term stability (**a**) and reusability stability (**b**) of the bioelectric activity sensor, (the concentration of Cu^2+^ used is 0.05 mM); (**c**) The impact of microbial contamination on the sensor; (**d**) Detection results of the sensor for both Cu^2+^ and Zn^2+^.

The stability of the bioelectroactive hydrogel sensor was investigated. As shown in Figure 6a, after storing the sensor at 4 °C for 5 days, it was still able to achieve over 90% of the current suppression rate. After storage for more than 6 days, the current response remained at 80%, and the detection performance began to decline significantly. This indicates that the detection platform has excellent storage stability, allowing for storage up to 5 days while maintaining reliable detection responses. Further investigation was conducted on the operational stability of the sensor. As shown in Figure 6b, after six consecutive uses, the sensor’s current output remained at a high level, with the response performance maintaining around 90%. After nine consecutive detections, the response decreased to approximately 70%. The sensor exhibited a significant performance decline after six detections, which may be attributed to the irreversible effect of Cu^2+^ on the activity of GOx. This suggests that the sensor has a certain level of reusability.

Subsequently, the antimicrobial resistance of the sensor was investigated. Antimicrobial experiments were conducted using Gram-positive bacteria (*Bacillus subtilis* and *Staphylococcus aureus*) and Gram-negative bacteria (*Escherichia coli* and *Aeromonas hydrophila*). Bacteria at a concentration of 10^−7^ CFU/mL were added to Cu^2+^ solutions for detection. The impact of bacterial contamination on the sensor’s response performance was analyzed by comparing the current response values with those of the normal sample. As shown in Figure 6c, the addition of other microorganisms to the sample did not affect the sensor’s current response compared to the uncontaminated sample. The sensor response remained between 93% and 96%, indicating that microbial contamination does not have a significant effect on the bioelectroactive hydrogel sensor in the short term.

In order to investigate the detection effect of this hydrogel sensor in real samples, the spiked recovery of Cu^2+^ in real wastewater by this sensor was further tested. Real livestock wastewater was taken from a pig farm in Huai’an, Jiangsu Province, to which Cu^2+^ solutions with concentrations of 1 mM, 2 mM, 5 mM, and 10 mM were added and then detected, respectively. Table S2 shows the results of the spiked recoveries of the wastewater by the sensor, and the obtained spiked recoveries ranged from 88% to 106.5%, indicating that the hydrogel sensor has potential for application in the detection of heavy metal ions in real wastewater.

Finally, the selectivity of the sensor for heavy metal ions was investigated. As shown in Figure 6d, Zn^2+^ also exhibited an inhibitory effect on GOx activity, causing a decrease in the oxidation current. The suppression level (23.3%) was slightly higher than that observed for Cu^2+^ (16.5%) In addition, the ability of the system to simultaneously detect Cu^2+^ and Zn^2+^ was investigated. It was found that adding equal concentrations of Cu^2+^ and Zn^2+^ resulted in a greater current suppression (49.9%) than the suppression caused by either metal ion alone, and it was higher than the sum of the suppression rates observed when detecting each ion individually. This may be because the inhibition of enzymatic reactions by heavy metal ions is not a selective process. As a result, this method lacks specific detection capability for heavy metal ions in practical applications. However, the system can be developed into a detector for assessing the total heavy metal content in water samples based on the inhibitory effect of heavy metals on enzymatic reactions.

## 4. Conclusions

In summary, this research successfully synthesized a bioelectroactive hydrogel sensor using the GOx surface display system with MR-1. The hydrogel electrode demonstrated high cell viability and maintained high GOx activity. The electrochemical detection platform successfully detected glucose and Cu^2+^, with the recovery rate of Cu^2+^ in real livestock and poultry wastewater ranging from 88% to 106.5%. Further investigation was conducted on the effect of Zn^2+^ on the detection results. The results indicated that Zn^2+^ also inhibits enzyme activity, and the presence of both Cu^2+^ and Zn^2+^ in the solution leads to a stronger inhibitory effect on the enzyme. This suggests that the sensor could be developed for assessing the overall heavy metal content in water samples. Moreover, factors such as the reduction level of graphene oxide and water content in the hydrogel may influence the mechanical stability, ionic conductivity, and molecular diffusion kinetics of the hydrogel sensor, which remains to be optimized in the future to further improve the performance of the sensing system. Overall, the sensor developed here combines the high specificity of biological recognition with the excellent physicochemical properties of nanomaterials and is expected to enable trace, rapid, and accurate detection of heavy metal ions in the environment.

## Data Availability

The data are contained within the article.

## Supplementary Materials

The following supporting information can be downloaded at: https://www.mdpi.com/article/10.3390/bios15060341/s1, Figure S1: The map of pYD1-GOx plasmid. Figure S2: The result of enzyme digestion validation of pYD1 plasmid, in which bands 1 and 2 are the result of plasmid enzyme digestion band 3 is the result of Maker. Figure S3: PCR validation results of pYD1-GOx plasmid, where the templates in bands 1–4 are pYD1-GOx plasmid extracted from *E. coli*; pYD1-GOx plasmid extracted from recombinant yeast; recombinant yeast broth as well as wild yeast broth, respectively, and band 5 is Maker. Figure S4: Optical diagram of the formation process of capillary hydrogel electrode at (a) 0 h and (b) 24 h; (c) zoomed-in red section of (b); (d) graphene hydrogel electrode. Figure S5: Cu^2+^ was introduced into the capillary-based sensor through a controlled loading process. Table S1: Comparison of performance parameters of typical sensors used for heavy metal ion detection. Table S2: Spiked recovery of the hydrogel sensor. References [37,38,39,40,41,42,43,44,45,46] are cited in the supplementary materials.
