# Supplementary material for: A Bioelectric Active Hydrogel Sensor for Trace Detection of Heavy Metal Ions in Livestock and Poultry Farm Wastewater
Source: Biosensors (Basel). 2025 May 29;15(6):341. doi: 10.3390/bios15060341 (PMC12190239; doi:10.3390/bios15060341)
Supplement: Supplementary file 1 [file biosensors-15-00341-s001.zip › biosensors-3616823-supplementary.pdf]

Supporting Information for

A Bioelectric Active Hydrogel Sensor for Trace Detection of Heavy Metal Ions in Livestock and Poultry Farm Wastewater

Heng-Chi Liu†, Jia-Xin Du†, Jie Wang, Junying Liu, Luyu Yang*, Yang-Chun Yong*

Institute for Energy Research, School of Environment and Safety Engineering, Jiangsu University, 301 Xuefu Road, Zhenjiang 212013, China

*Corresponding author, E-mail: yangluyu@ujs.edu.cn (Luyu Yang); ycyong@ujs.edu.cn (Yang-Chun Yong)

†These authors contributed equally to this work.

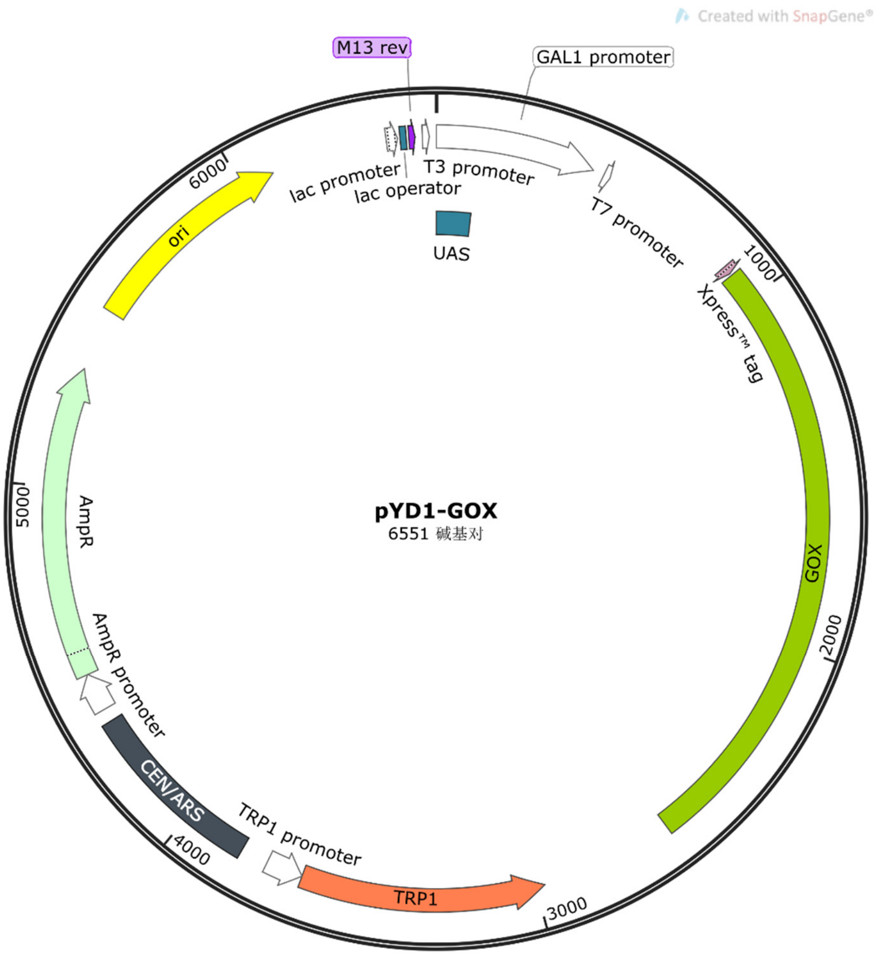

Figure S1. The map of pYD1-GOx plasmid

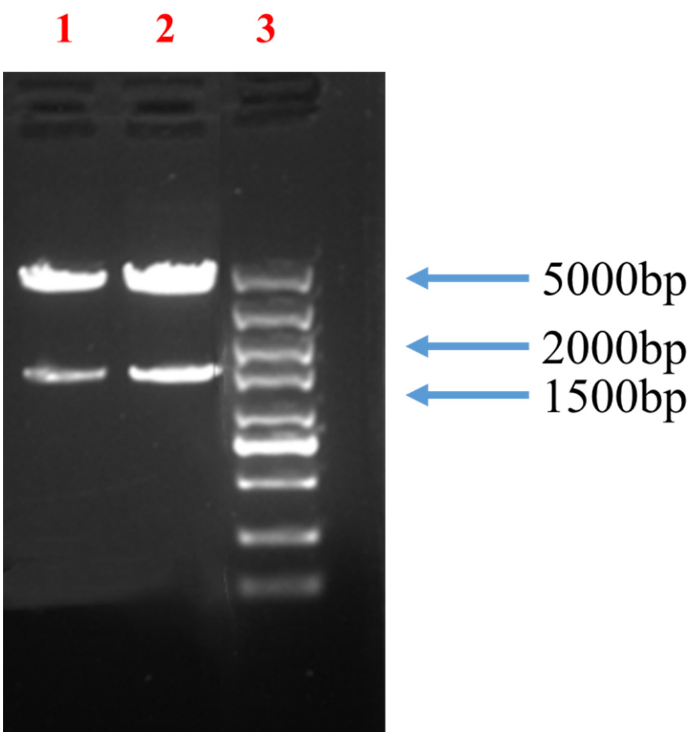

Figure S2. The result of enzyme digestion validation of pYD1 plasmid, in which bands 1 and 2 are the result of plasmid enzyme digestion band 3 is the result of Maker

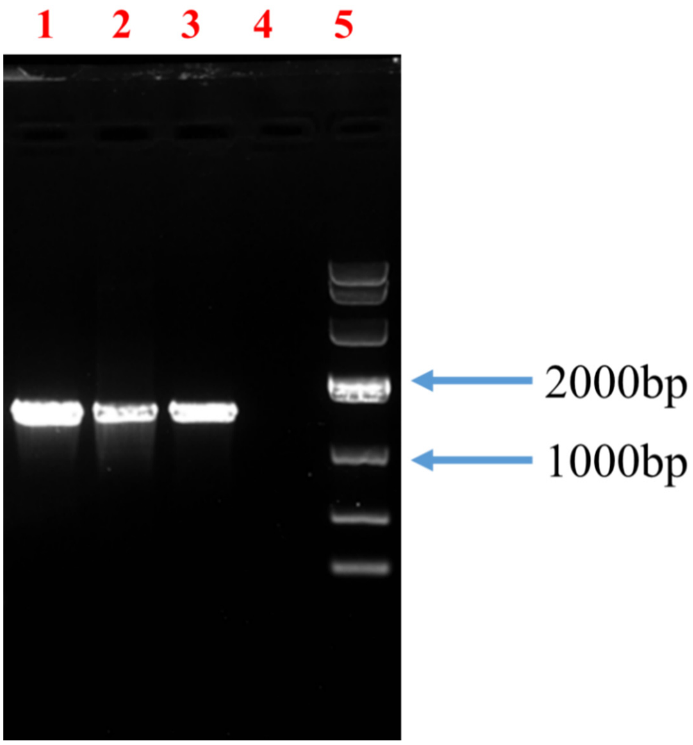

Figure S3. PCR validation results of pYD1-GOx plasmid, where the templates in bands 1-4 are pYD1-GOx plasmid extracted from E. coli; pYD1-GOx plasmid extracted from recombinant yeast; recombinant yeast broth as well as wild yeast broth, respectively, and band 5 is Maker

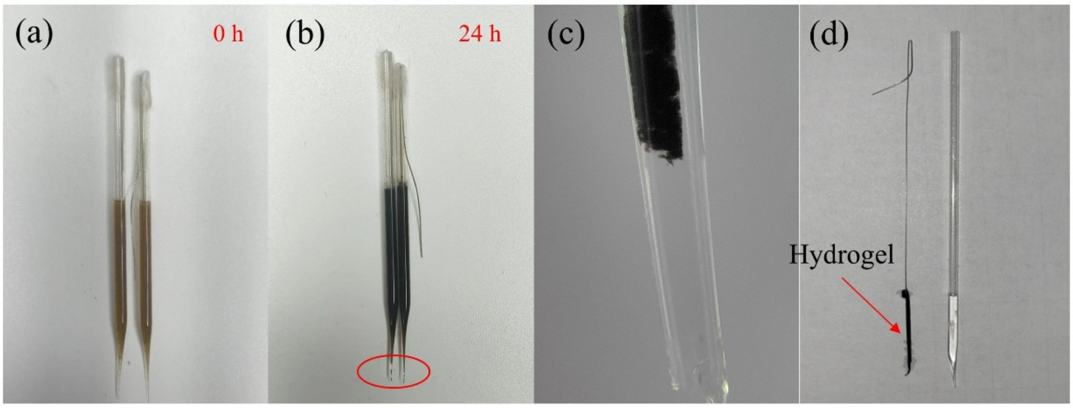

Figure S4. Optical diagram of the formation process of capillary hydrogel electrode at (a) 0 h and (b) 24 h; (c) zoomed in red section of (b); (d) graphene hydrogel electrode

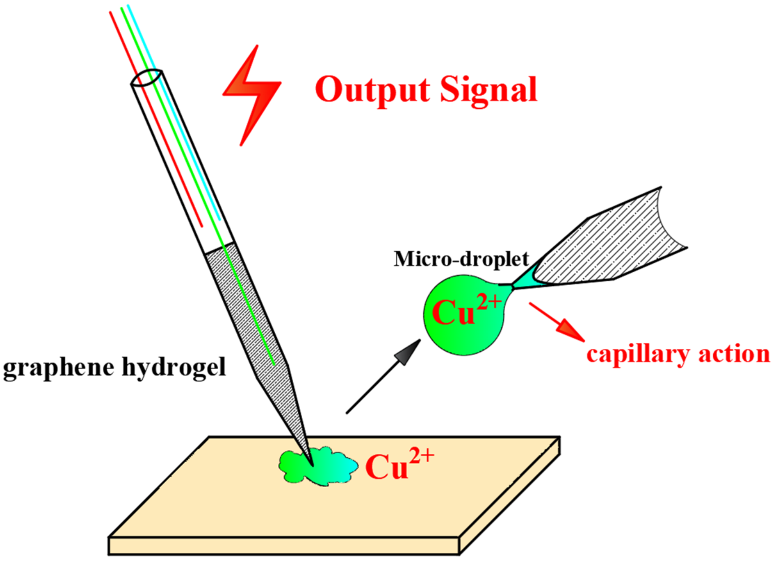

Figure S5. Cu^{2+} was introduced into the capillary-based sensor through a controlled loading process

Table S1. Comparison of performance parameters of typical sensors used for heavy metal ion detection.

Materials	Method	Target	LOD (μM)	Sample	Ref.
Bioelectric Active Hydrogel	DPV	Cu^{2+}	17	Wastewater	This work
MFC	Direct response	Cu^{2+}	78	Wastewater	[1]
SMFC	Direct response	Cu^{2+}	196	Wastewater	[2]
Glutathione modified SPE with carbon nanofiberelectrode	CV	Cu^{2+}	47.2	Wastewater	[3]
Chitosan/PANi–Bi nanoparticle@graphene oxide multi-walled carbon nanotubes (CS/PANi–Bi NP@GO–MWCNT)	DPV	Cu^{2+}	15.6	Tap water	[4]
Beta galactosidase enzyme (β-gal) on bare goldelectrode	CV	Cd^{2+}	61.8	River water	[5]
Gold Nanorod Chains	Dynamic Light Scattering	Pb^{2+}	25	Standard solution	[6]
GO-IIP-IDE	Thermal polymerization	Hg^{2+}	4.98	River water	[7]
Bi-CP-GCE	SWASV	Cd^{2+}	25	Standard solution	[8]
Quartz Crystal Microbalance Sensor	CA	Cu^{2+}	157	Wastewater	[9]
Polyethylene glycol-capped gold nanoparticles (PEG-AuNPs)	SWASV	As^{3+}	38.7	Tap water	[10]

Table S2. Spiked recovery of the hydrogel sensor

Sample	Spiked (mM)	Detected by biosensor (mM)	Recovery
1	1	0.88	88%
2	2	2.13	106.5%
3	5	4.47	89.4%
4	10	9.15	91.5%

References

1. Shen, Y. J.; Wang, M.; Chang, I. S.; Ng, H. Y., Effect of shear rate on the response of microbial fuel cell toxicity sensor to Cu(II). *Bioresour. Technol.* **2013,** 136, 707-710.
2. Liu, L.; Lu, Y.; Zhong, W. H.; Meng, L.; Deng, H., On-line monitoring of repeated copper pollutions using sediment microbial fuel cell based sensors in the field environment. *Sci. Total Environ.* **2020,** 748.
3. Pérez-Ràfols, C.; Serrano, N.; Díaz-Cruz, J. M.; Ariño, C.; Esteban, M., Glutathione modified screen-printed carbon nanofiber electrode for the voltammetric determination of metal ions in natural samples. *Talanta* **2016,** 155, 8-13.
4. Bao, Q. W.; Li, G.; Yang, Z. C.; Pan, P.; Liu, J.; Li, R. R.; Wei, J.; Hu, W.; Cheng, W. B.; Lin, L., In situ detection of heavy metal ions in sewage with screen-printed electrode-based portable electrochemical sensors. *Analyst* **2021,** 146, 5610-5618.
5. Fourou, H.; Zazoua, A.; Braiek, M.; Jaffrezic-Renault, N., An enzyme biosensor based on beta-galactosidase inhibition for electrochemical detection of cadmium (II) and chromium (VI). *Int. J. Environ. Anal. Chem.* **2016,** 96, 872-885.
6. Durgadas, C. V.; Lakshmi, V. N.; Sharma, C. P.; Sreenivasan, K., Sensing of lead ions using glutathione mediated end to end assembled gold nanorod chains. *Sens. Actuators, B-Chem.***2011,** 156, 791-797.
7. Yasinzai, M.; Mustafa, G.; Asghar, N.; Ullah, I.; Zahid, M.; Lieberzeit, P. A.; Han, D.; Latif, U., Ion-Imprinted Polymer-Based Receptors for Sensitive and Selective Detection of Mercury Ions in Aqueous Environment. *J. Sens.* **2018,** 2018.
8. Xuan, X.; Park, J. Y., A miniaturized and flexible cadmium and lead ion detection sensor based on micro-patterned reduced graphene oxide/carbon nanotube/bismuth composite electrodes. *Sens. Actuators, B-Chem.* **2018,** 255, 1220-1227.
9. Cao, Z.; Guo, J. K.; Fan, X.; Xu, J. T.; Fan, Z. Q.; Du, B. Y., Detection of heavy metal ions in aqueous solution by P(MBTVBC-co-VIM)-coated QCM. *Sens. Actuators, B-Chem.* **2011,** 157, 34-41.
10. Shalvi; Kumar, N.; Verma, K. L.; Jain, V. K.; Nagpal, S., Correction to: Integrated

device for colorimetric detection of arsenite using polyethylene glycol capped gold nanoparticles—Lab-on-chip. *J. Toxicol. Environ. Health Sci.* **2021,** 13, 425-427.
